# Identification of prognostic markers related to homologous recombination deficiency in cholangiocarcinoma using CoxBoost and LASSO machine learning techniques

**DOI:** 10.3389/fimmu.2026.1615657

**Published:** 2026-01-21

**Authors:** Yan Liu, Cheng Zhou, Tianhao Shen, Xue Yu, Qiuying Li, Tinghui Jiang, Wei Li, Yongqiang Zhu

**Affiliations:** 1 Oncology Intervention Department, Putuo Hospital, Shanghai University of Traditional Chinese Medicine, Shanghai, China; 2 Hepatopancreatobiliary Surgery Department, Putuo Hospital, Shanghai University of Traditional Chinese Medicine, Shanghai, China

**Keywords:** cholangiocarcinoma, genetic variations, homologous recombination deficiency, immune infiltration, multiple machine learning

## Abstract

**Background:**

Cholangiocarcinoma (CHOL) is a highly aggressive malignancy with a poor prognosis. Homologous recombination deficiency (HRD) is associated with genomic instability and cancer progression, making it a potential therapeutic target. The aim of this study is to develop novel potential prognostic biomarkers and construct an HRD-based prognostic risk prediction model for CHOL to enhance clinical precision medicine.

**Methods:**

We analyzed HRD across cancers using multiple datasets including TCGA-CHOL, TCGA-LIHC, GDC TARGET-OS, and IMvigor210. HRD scores were calculated using data from Thorsson et al. and the maftools R package was used for mutation data visualization and tumor mutational burden (TMB) calculation. Differential gene expression analysis identified HRD-related genes, validated in tumor and adjacent non-tumor tissues using RT-PCR. 10 machine-learning algorithms including RSF, LASSO, GBM, Survival-SVM, SuperPC, Ridge, plsRcox, CoxBoost, Stepwise Cox, Enet were selected to construct a prognostic model and validated in the E-MTAB-6389 and GSE107943. Among them, RSF, LASSO, CoxBoost and Stepwise Cox have the functions of dimension reduction and variable screening.

**Results:**

Comparative analysis demonstrated significant associations between HRD scores and genomic instability markers. High HRD scores independently predicted poorer overall survival (log-rank p = 0.043) and progression-free interval (log-rank p = 0.028). Immune infiltration analysis revealed higher levels of active B cells and regulatory T cells in the low-risk group, suggesting differential immune landscapes between risk groups. We identified the CoxBoost and LASSO algorithms as the optimal combination for creating a CoxBoost+ LASSO prognostic model. Using this model, we identified six genes (ANXA2P1, BBOX1, KLHL33, MN1, OR51A4, and TRDN) with significant differential expression.

**Conclusions:**

Our HRD-based prediction model offers a reliable tool for CHOL prognosis, suggesting new potential for six candidate genes as prognostic biomarkers. It highlights potential therapeutic targets and drug sensitivities, providing new insights into personalized treatment strategies for CHOL management.

## 1 Introduction

Cholangiocarcinoma (CHOL) is a highly malignant tumor of the digestive tract characterized by strong heterogeneity and poor prognosis. The 5-year survival rate is approximately 5%, and the median survival time is less than 1 year (1). In recent years, the incidence of CHOL has increased globally, with an annual incidence of 125/10,000 per year in our country (2–4). Current treatment methods, including surgical resection, chemotherapy, and radiotherapy, have significant limitations. Surgical resection is only suitable for early-stage patients and carries a high risk of postoperative recurrence and metastasis. Chemotherapy and radiotherapy, while potentially improving survival, exhibit considerable inter-individual variability in efficacy and often come with severe side effects (5, 6). These limitations necessitate the exploration of more precise and efficient treatment methods. The mechanisms underlying the occurrence and development of CHOL are complex, involving the abnormal regulation of multiple genes and pathways. Genetic mutations, epigenetic changes, and abnormal activation of signaling pathways collectively contribute to CHOL progression. However, the specific relationships and regulatory networks among these mechanisms are not fully understood, which hinders the development of targeted therapies. Additionally, CHOL lacks effective molecular prognostic markers. Traditional clinicopathological indicators such as tumor size, degree of differentiation, and lymph node metastasis have limited accuracy in predicting patient prognosis. Identifying molecular markers that can accurately assess CHOL prognosis is therefore crucial for guiding clinical treatment and improving patient survival.

Homologous recombination deficiency (HRD) is a genetic condition that impairs the ability of cells to repair double-stranded DNA breaks, leading to genomic instability and increased susceptibility to certain cancers. HRD has been extensively studied in breast and ovarian cancers and has been shown to predict responses to PARP inhibitors and platinum-based chemotherapy (7). The ATLAS trial (NCT03397394) showed PARPi has limited efficacy in treating HRD-positive urothelial carcinoma (8). Interim results of the ORCHID trial (NCT03786796) indicated a disease control rate (DCR) of 18% and objective response rate (ORR) of 9% in patients with metastatic renal cell carcinoma (RCC) adopting Olaparib (9). Recent studies suggest that HRD may also play a role in CHOL (10). Mutations in BRCA1/2 and other HRD-related genes have been identified in a subset of patients with CHOL, indicating that HRD may be a potential therapeutic target for this malignancy (11).

This study seeks to improve the diagnosis and treatment of CHOL by systematically screening and validating a new cluster of prognostic biomarkers based on omics features. We developed an innovative risk stratification model based on HRD to address three key issues in current clinical practice. First, considering the high recurrence rate and the absence of effective early warning indicators in CHOL patients after surgery, we identified biological targets for prognosis. Second, the HRD-based prognostic model will address the limitations of the traditional TNM staging system. It will create a quantifiable molecular classification system by integrating various dimensions of information, including genomic instability, epigenetic modifications, and tumor microenvironment heterogeneity. It provides a decision-making basis for clinicians to choose the timing of surgery, optimize adjuvant chemotherapy regimens, and evaluate the applicability of immune checkpoint inhibitors. Thirdly, the establishment of an individualized prognostic risk scoring system can accurately identify high-risk recurrence groups and guide the application of preoperative neoadjuvant therapy, while avoiding overtreatment of low-risk patients, significantly improving the quality of life of patients and reducing medical costs. This study aims to establish a comprehensive translational medicine paradigm that includes biomarker discovery, molecular mechanism analysis, clinical model construction, and treatment strategy optimization. This approach will provide new molecular diagnostic tools for accurately classifying CHOL and will shift the treatment of biliary tract cancer from empirical medication to individualized therapies driven by molecular characteristics.

## 2 Materials and methods

### 2.1 Data collection

This research was carried out strictly in accordance with the predetermined process. For details, please refer to **Supplementary Figure 1**. The patient cohorts and datasets used for the analysis include the following parts: (1) The TCGA-CHOL (The Cancer Genome Atlas Cholangiocarcinoma) dataset, derived from the UCSC Xena platform (12). A total of 45 patients with cholangiocarcinoma were included in their transcriptome sequencing data, combined with their publicly available clinical information, including age (range 32–85 years, median 62 years), gender (23 males and 22 females), survival time and survival status. The inclusion criteria are as follows: a clear pathological diagnosis of CHOL, complete gene expression and clinical follow-up information; The exclusion criteria were: cases with missing key clinical data or follow-up data. Ultimately, 36 patients met the analysis criteria. The patients in this cohort were mainly from North America and Europe. (2) Datasets such as TCGA-LIHC (hepatocellular carcinoma), GDC TARGET-OS (osteosarcoma), and IMvigor210 (immunotherapy for bladder cancer) were respectively used for the analysis of pan-cancer HRD. They were all downloaded from public databases. For specific inclusion and exclusion criteria, please refer to **Supplementary Table 1**. (3) The external validation cohort included E-MTAB-6389 (from the Bio-Studies database) (13) and GSE107943 (from the GEO database) (14), with a total of 75 patient samples of cholangiocarcinoma included. All cases were patients with a clear diagnosis and complete clinical information, and those with other malignant tumors or lacking follow-up data were excluded. The specific sources, enrollment times, geographical distributions, and demographic characteristics of each dataset (for details, see **Supplementary Table 1**) are provided to enhance the reproducibility and data transparency of the study.

### 2.2 Assessment of HRD and calculation of neoantigen score

The HRD score was defined as the unweighted sum of the loss of heterogeneity (LOH) (15), telomere allele imbalance (TAI) (16), and large-scale transition (LST) scores (17, 18). Neoantigen load, predicting peptides binding to major histocompatibility complex (MHC) proteins, derived from RNA-seq HLA typing. Neoantigen burden was quantified by single nucleotide variants (SNVS) and insertion/deletion (indel) mutations. Values for HRD, neoantigen load (SNV and Indel), and mutation rate were collated from the pan-cancer Atlas study by Thorsson et al. (19). We compared genome alteration fractions, mutation counts, and MSI sensor scores between the top and bottom 20% HRD score patients in CHOL. TCGA-CHOL patients were stratified into high and low HRD subgroups by median HRD score (**Supplementary Table 2**).

### 2.3 Characteristic genes associated with HRD score

We used the Limma (V3.56.2) R package (20) to analyze the differences between HRD high-risk and low-risk patients. We set cutoffs at log2fold change > 1 and P < 0.05. Genes with log2 fold change > 1 were considered highly expressed in the HRD group, while those with log2 fold change < -1 were considered low-expressed. We visualized these differences using volcano plots, ranking maps, and a heat map. Spearman’s correlation analysis was performed to explore gene correlations.

### 2.4 Multi-machine learning for feature gene screening and prognostic model construction

To construct a robust prognostic model for CHOL-HRD, we followed a multi-step approach: (1) First, we integrated 10 classical algorithms: Random forest (RSF), least absolute shrinkage and selection operator (LASSO), gradient boosting machine (GBM), Survival support vector machine (survival-SVM), supervised principal component (SuperPC), ridge regression (ridge), Cox Partial least squares regression (plsRcox), CoxBoost, Stepwise Cox, and elastic network (Enet). Among them, RSF, LASSO, CoxBoost, and Stepwise Cox have dimensionality reduction and variable screening functions, leading to 37 machine-learning algorithm combinations. (2) Using TCGA-CHOL as the training cohort, we applied these algorithm combinations to screen key genes and construct a prognostic model based on previously identified feature genes. (3) We validated the model using two test cohorts (E-MTAB-6389 and GSE107943), calculating the CHOL-HRD risk score based on features obtained from the training cohort. Based on the average C-index of the two test cohorts, we selected the best prognostic model for CHOL-HRD and calculated the risk score. Patients were divided into high-risk and low-risk HRD groups based on the median score. Survival and multivariate Cox analyses were used to evaluate the independent prognostic significance of the risk score.

### 2.5 Comparison of HRD score with other signatures

To further evaluate the predictive value of the HRD Score, we compared it with previously reported CHOL biomarker prognostic models (21–25). The predictive power of the other biomarkers was compared with that of the HRD Score in the two test cohorts (E-MTAB-6389 and GSE107943).

### 2.6 Prognostic power of HRD score in pan-cancer

Given the importance of HRD in cancer development, we explored whether the HRD Score has a stable prognostic effect on other types of tumors. Based on the overall survival of patients in the TCGA-LIHC, TCGA-OV, and TARGET-OS datasets, we used the survival R package (https://CRAN.R-project.org/package=survival) for survival analysis to verify the prognostic efficiency of the HRD Score.

### 2.7 GSEA enrichment analysis

We performed Gene Set Enrichment Analysis (GSEA) using the clusterProfiler 4.8.2 R package (26), calculating normalized enrichment scores for each gene set. The gene set “c2.cp.kegg.symbols.gmt” from the MSigDB database was used for GSEA to evaluate the impact of high- and low-risk groups on tumor KEGG pathways, with FDR < 0.25 considered significant.

### 2.8 Mutated gene oncogenic pathways, TMB, and MSI analysis

To analyze the Single Nucleotide Polymorphisms (SNPs) in different risk score groups of TCGA-CHOL patients, maftools 2.16.0 package was used to analyze the frequently mutated genes in the high and low risk groups. To evaluate the interactions between drugs and gene mutations, the drugInteractions function was used for analysis. The goal was to identify genetic mutations associated with susceptibility or resistance to specific drugs. In addition, biological oncogenic pathway analysis was performed on the mutation data to understand which biological oncogenic pathways are affected by gene mutations. We used the OncogenicPathways and PlotOncogenicPathways functions to achieve this goal. Meanwhile, we calculated the TMB data of CHOL patients through the maftools 2.16.0 R package. CHOL, patients with MSI - Sensor data is from cBioportal database (https://www.cbioportal.org).

### 2.9 Immune infiltration and differential analysis of immunomodulators

The CIBERSORT (https://cibersort.stanford.edu/) algorithm evaluated immune cell infiltration in different CHOL sample datasets. Differences in immune cell infiltration among disease subgroups were tested using the Wilcoxon test, with p < 0.05 considered statistically significant. We also assessed the differential expression of Immune Checkpoint Proteins (ICP) and Immunogenic Cell Death (ICD) modulators between high- and low-risk groups. The “estimate” R package was used to analyze differences in tumor immune score, stromal score, and tumor purity.

### 2.10 Development and validation of potential therapeutic agents

We used the Cancer Treatment Response Portal (CTRP, https://portals.broadinstitute.org/ctrp/) and the PRISM dataset to identify potential drugs for CHOL patients in the high-risk HRD group. Following the protocol by Yang et al. (27) 2021: (1) We obtained drug sensitivity data for CCLs from the CTRP and PRISM datasets and expression data from the Cancer Cell Line Encyclopedia (CCLE) database. (2) The CTRP and PRISM datasets provided AUC values, where lower AUC values indicate increased sensitivity to the compound. (3) Using the Wilcoxon rank-sum test, we performed a differential analysis of drug response between high- and low-risk groups based on the HRD Score. We set a threshold of log2FC > 0.03 to identify compounds with low AUC values in the high-risk HRD group. (4) Next, we used Spearman’s correlation to further screen compounds with a negative correlation coefficient between the Area Under the Curve (AUC) value and the HRD Score (threshold R < -0.1). (5) Finally, we identified potential drugs for high-risk HRD patients by intersecting the compounds obtained from steps (3) and (4). Furthermore, we used CMap to validate the results obtained from the CTRP and PRISM databases based on differential expression profiles.

### 2.11 Molecular docking

To identify potential drugs with significant associations with CHOL, we performed a comprehensive analysis using CTRP, PRISM, and Cmap. We downloaded the molecular structures of the drugs from PubChem (https://pubchem.ncbi.nlm.nih.gov/) and the target proteins from the Protein Data Bank (PDB; http://www.rcsb.org/). The connection between potential drugs and targets was visualized using CB-Dock2 (https://cadd.labshare.cn/cb-dock2/php/index.php).

### 2.12 Clinical sample collection

This study also included clinical samples from 6 patients with primary intrahepatic cholangiocarcinoma (ICC) diagnosed by pathology. All of them underwent surgical resection in the Department of Interventional Oncology of Putuo Hospital affiliated with Shanghai University of Traditional Chinese Medicine from January 2023 to June 2023. The patients’ ages ranged from 63 to 78 years old (median 70.5 years old), including 4 males and 2 females. The inclusion criteria were: (1) ICC was clearly diagnosed by imaging and/or histology before the operation; (2) No history of neoadjuvant therapy, initial surgical resection; (3) No history of other malignant tumors or serious underlying diseases; (4) Postoperative pathology confirmed ICC, and both the tumor and the paired adjacent tissues could be obtained. The exclusion criteria are: (1) Previous or combined with other malignant tumors; (2) Receive radiotherapy, chemotherapy or immunotherapy/targeted therapy before the operation; (3) There are serious complications that affect the quality of the samples. All patients signed the informed consent form, and the sample collection was approved by the hospital ethics committee (Approval Number: PTEC-A-2024-7-1). Tumor tissues and paired adjacent non-tumor tissues were collected from each patient respectively. They were immediately frozen in liquid nitrogen after the operation and stored at -80°C for subsequent molecular detection and analysis. Detailed demographic and clinical information can be found in **Supplementary Table 2**.

### 2.13 Real-time PRC analysis

Total RNA was extracted from cancerous and adjacent tissues using TRIzol reagent (Invitrogen) according to the manufacturer’s protocol. cDNA was synthesized with the RevertAid First Strand cDNA Synthesis Kit (Thermo Fisher Scientific, Waltham, MA, USA). Real-time PCR (RT-PCR) was performed using the Maxima SYBR Green/ROX qPCR Master Mix under the following thermal cycling conditions: 1) 95°C for 10 min; 2) 95°C for 15 s, then 60°C for 45 s, repeated for 40 cycles; 3) 95°C for 15 s; 4) 60°C for 1 min; 5) 95°C for 15 s; 6) 60°C for 15 s. Relative miRNA levels were analyzed using ABI Prism7300 SDS Software (Foster City, CA, USA). The primer sequences used for the analysis are shown in **Supplementary Table 3**.

### 2.14 Statistical analysis

All statistical analyses were performed using R software (version 4.3.1 and 3.6.0). The Wilcoxon rank-sum test was used to compare differences between two groups, while the Kruskal–Wallis test was applied to evaluate differences among more than two groups. Spearman’s correlation test was employed for correlation analysis. p < 0.05 (two sides) were considered statistically significant.

## 3 Results

### 3.1 Study procedure flow

The high-risk HRD group of CHOL is characterized by genomic instability, with significant differences in genomic alterations and MSI observed in TCGA-CHOL. A longer OS indicates that the HRD score can serve as a reliable prognostic biomarker for CHOL. We performed 240 identified candidate genes through differential gene screening. The CoxBoost and Lasso combination, with the highest average C-index among 37 machine-learning algorithms, was selected as the best model and validated using two external datasets. Finally, multi-omics analysis, including genome, proteome, metabolome, and single-cell analysis, was performed for cancer screening, diagnosis, drug response, and prognosis prediction (**Supplementary Figure 1**).

### 3.2 HRD score predicts CHOL

**Figure 1:**
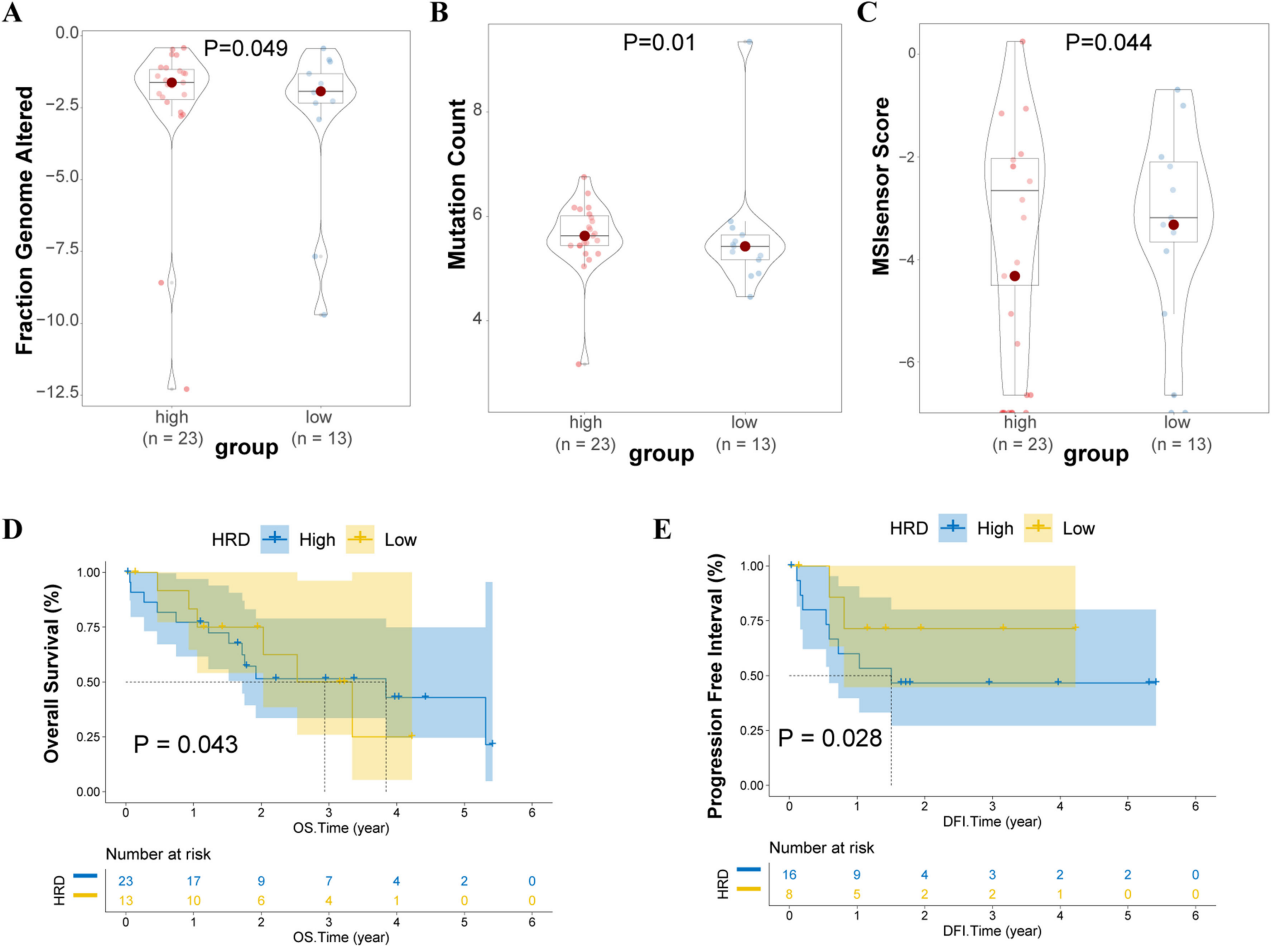
Significance of HRD Score. **(A-C)** Violin plots of fraction genomic alteration, mutation counts, and MSI in high and low HRD score groups; **(D, E)** OS and PFS of patients with high and low HRD scores in TCGA-CHOL cohort.

To explore the correlation between HRD score and genomic instability, patients with CHOL were divided into high- and low-scoring groups based on the median HRD score. Significant differences were observed in genomic alterations (**Figure 1A**), somatic mutation count (**Figure 1B**), and MSI (**Figure 1C**) between the groups. Additionally, TAI (Supplementary **Figure 2A**), LST (Supplementary **Figure 2B**), and LOH (**Supplementary Figure 2C**) were higher in the high HRD score group. Survival analysis showed significant differences in OS (log-rank p = 0.043, HR (95%Cl): 2.0108 (1.415-2.858), high: 181, low: 182, **Figure 1D**) and PFS (log-rank p = 0.043, HR (95%Cl): 3.5163 (1.547-7.995), high: 42, low: 43, **Figure 1E**) between the groups, with worse prognosis in the high HRD score group, suggesting HRD score as a good prognostic indicator.

**Figure 2:**
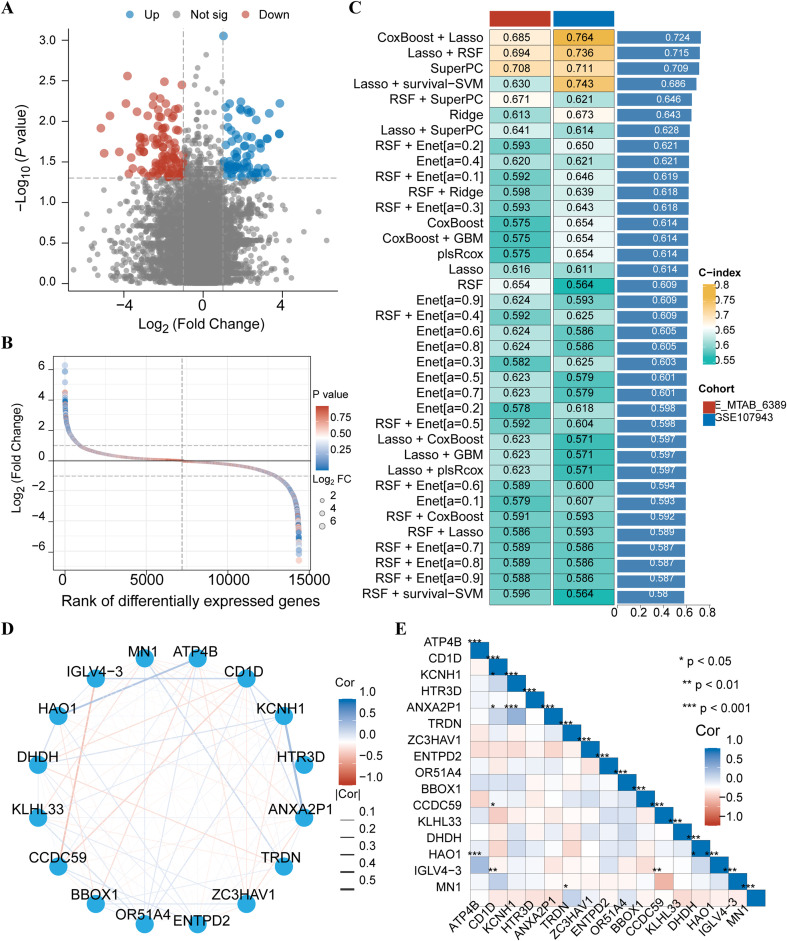
Selection of Key Genes and Prognostic Model. **(A)** Volcano map of differentially expressed genes between high and low HRD expression groups; **(B)** LogFC value distribution of differentially expressed genes in high and low HRD expression groups; **(C)** C-index heat map of CoxBoost and Lasso combined prognostic model in validation sets E-MTAB-6389 and GSE107943; **(D)** Correlation network diagram of 16 important genes screened by CoxBoost model; **(E)** Correlation heat map of 16 important genes screened by CoxBoost model.

### 3.3 Construction of multi-machine learning models based on HRD feature genes

Identifying key HRD-related genes is crucial for developing CHOL predictive biomarkers and understanding the source of HRD. To identify these key genes, we analyzed the differences between the high and low-expression groups. We identified 240 candidate genes (**Figure 2A**). The distribution of the logFC values for these genes is shown in **Figure 2B**. We then focused on constructing the best prognostic model. The combination of CoxBoost and Lasso, with the highest average C index (0.724) among the 37 machine-learning algorithms, was selected as the final model (**Figure 2C**). In this combined model, 17 significant genes were screened, and 16 were selected for model construction after multivariate analysis. Correlation analysis of these 16 genes showed multiple strong correlations (**Figures 2D, E**).

### 3.4 Establish a prognostic model using CoxBoost and Lasso machine-learning

**Figure 3:**
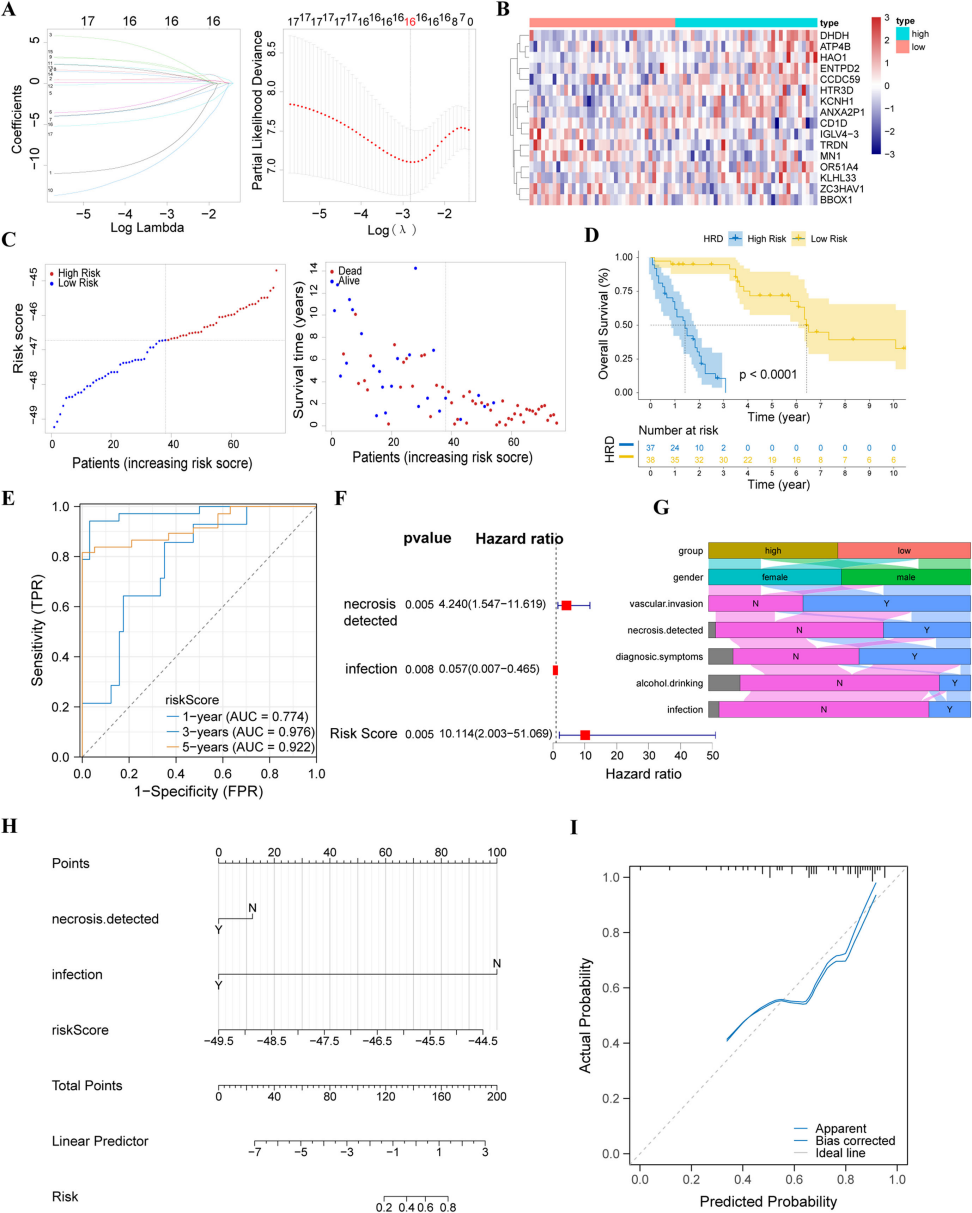
Development and Validation of a HRD Risk Model. **(A)** Lasso regression analysis showing 16 variables corresponding to the optimal lamda value; **(B)** Heat map of characteristic gene expression in the Lasso model; **(C)** Risk score distribution and survival status of CHOL patients based on the Lasso model; **(D)** Kaplan–Meier curve for high and low-risk patients in the E-MTAB-6389 dataset; **(E)** Receiver operating characteristic (ROC) curve of the HRD risk score model (timeROC curve); **(F)** Multivariate Cox regression analysis evaluating the independent prognostic value of the HRD risk score (forest plot); **(G)** Sankey diagram showing the distribution of clinical characteristics in high and low-risk HRD groups; **(H)** Nomogram model for patient prognosis; **(I)** Calibration curve for the nomogram model.

Based on the C-index comparison of 37 machine-learning model combinations, the CoxBoost and Lasso algorithms were identified as the optimal combination for constructing an HRD-related prognostic prediction model for CHOL patients. From 240 key genes, 16 critical genes were selected to construct the LASSO prognostic model (**Figure 3A**). These genes’ median expression values were used to divide patients into subgroups for separate prognosis analysis. Seven genes (ANXA2P1, ATPB, BBOX1, KLHL33, MN1, OR51A4, and TRDN) exhibited significant prognostic differences (**Supplementary Figures 3A–G**). Using the penalty coefficients calculated by multivariate Cox analysis (**Supplementary Table 4**), we established a risk score by multiplying gene expression values by their corresponding coefficients. This allowed us to calculate the final risk score for each sample, dividing patients into high- and low-risk groups. A risk factor heat map was created based on patients’ risk scores and the expression values of the 16 key genes. In addition, a higher number of patients with high HRD scores died (**Figures 3B, C**).

The Kaplan–Meier curve showed a significantly worse prognosis for high-risk patients, with substantial survival differences between the high- and low-risk groups (**Figure 3D**). This finding was validated in two other datasets: TCGA-CHOL (**Supplementary Figure 2D**) and GSE107943 (**Supplementary Figure 2E**). Time ROC analysis showed that the area under the curve (AUC) for 1-, 3-, and 5-year survival predictions were 0.774, 0.976, and 0.922, respectively, demonstrating the HRD risk score model’s strong predictive performance (**Figure 3E**). Multivariate Cox Regression analysis showed that the HRD risk score serves as an independent prognostic factor for CHOL patients, similar to bile duct necrosis and infection (**Figures 3F, G**). According to these results, bile duct necrosis, infection, and the HRD risk score were incorporated into a nomogram for further prognosis prediction (**Figure 3H**). The diagnostic calibration curve indicated a good fit (**Figure 3I**).

### 3.5 Comparison of prognostic models and evaluation of generalization ability

**Figure 4:**
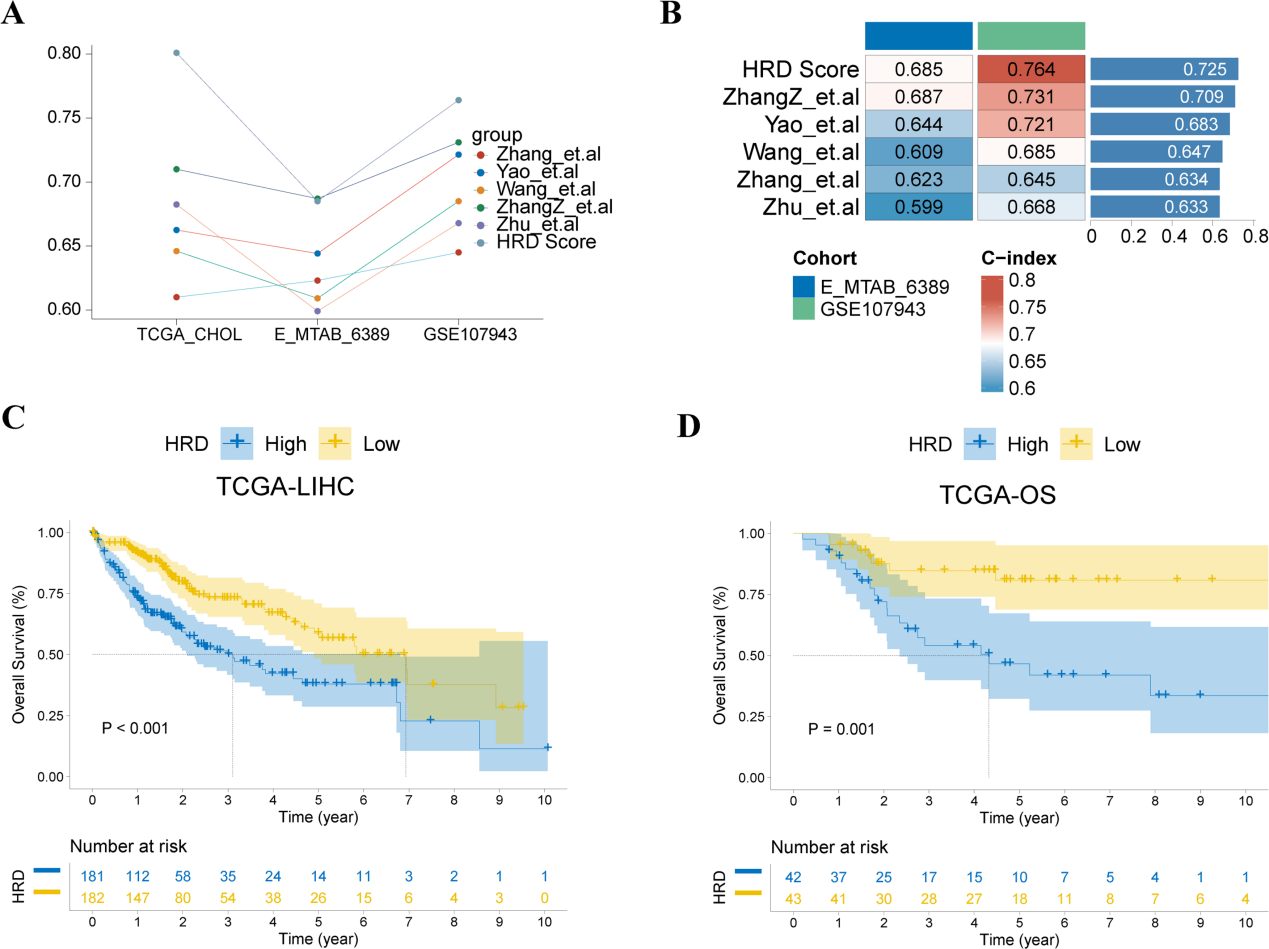
Pan-cancer ability assessment of HRD scores. **(A)** C-index distribution plot of HRD score prognostic prediction model and other published prognostic prediction models; **(B)** HRD score and C-index heat map of other published prognostic prediction models; **(C)** Kaplan-Meier curve of the hepatocellular carcinoma dataset TCGA-LIHC showing that the prognosis of high-risk patients is significantly worse (P < 0.001); **(D)** K-M curve of the TCGA-OS patients showing that the prognosis of the high-risk group is significantly worse (P = 0.001).

To validate the prognostic ability of the HRD score model, we compared its performance with that of published CHOL prognostic models. The results showed that the HRD score had high and stable predictive performance in the validation sets E-MTAB-6389 and GSE107943 (**Figure 4A**). The average C-index of the HRD score model was significantly better than that of the other models (**Figure 4B**). HRD significantly affects tumor progression not only in CHOL but also in other types of tumors. To explore the generalization of the prognostic value of the HRD score, we evaluated its performance in the TCGA hepatocellular carcinoma and TARGET-OS (osteosarcoma) datasets. The Kaplan–Meier survival curves showed that the HRD score had significant prognostic differences not only in CHOL patients but also in hepatocellular carcinoma (**Figure 4C**) and osteosarcoma (**Figure 4D**). There were significant prognostic differences between high and low HRD scores.

### 3.6 Analysis of pathway enrichment and genomic features between high- and low-risk HRD groups

**Figure 5:**
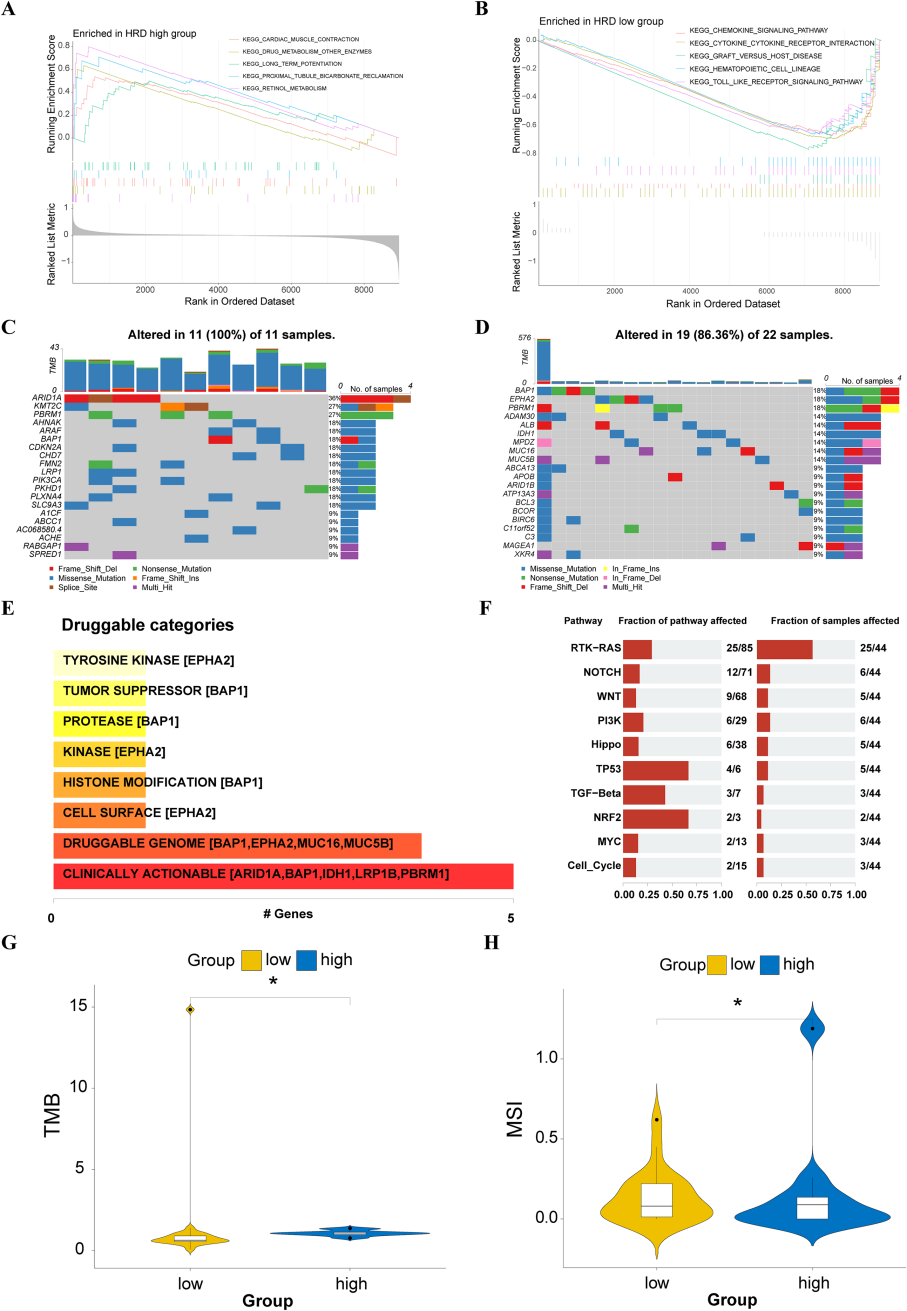
Genomic differences between high- and low-risk groups of HRD. **(A)** Enrichment of high-risk HRD group pathways. **(B)** Enrichment of low-risk HRD group pathways; **(C)** Mutational profiles of common tumorigenic drivers among patients in the high-risk group; **(D)** Mutational profiles of common tumorigenic drivers among patients in the low-risk group; **(E)** Enrichment bar chart of mutant gene patent drug pathway based on mutation data of CHOL patients; **(F)** Enrichment bar chart of carcinogenic pathways based on mutation data of CHOL patients; **(G)** Violin plot of the difference in tumor mutation burden between high and low-risk groups; **(H)** Violin plot of the difference in MSI between high and low-risk groups.

To investigate differences in signal pathway enrichment between high- and low-risk HRD groups, we performed GSEA on both groups. The top five gene pathways significantly enriched in the high-risk HRD group were cardiac muscle contraction, drug metabolism, long-term potentiation, proximal tubule bicarbonate induction, and retinol metabolism (**Figure 5A**). In the low-risk HRD group, the top five enriched pathways were the chemokine signaling pathway, cytokine receptor interaction, graft versus host disease, hematopoietic cell lineage, and Toll-like receptor signaling pathway (**Figure 5B**).

We further evaluated the effect of HRD score on genetic variants, including SNPs and copy number variations (CNVs), in patients with CHOL. Analysis of single-nucleotide mutations in common driver genes during tumorigenesis showed a higher mutation rate in the high-risk group compared to the low-risk group (**Figures 5C, D**). From mutation data in CHOL patients, we identified mutations associated with susceptibility or resistance to specific drugs, revealing strong links between mutated genes and multiple proprietary pathways (**Figure 5E**). Furthermore, we identified cancer pathways enriched with mutated genes in CHOL patients, with the RTK-RAS signaling pathway showing the highest enrichment (**Figure 5F**).

A differential analysis of the TMB (**Figure 5G**) and MSI scores (**Figure 5H**) demonstrated significantly higher genomic instability in the high-risk group. Although statistically significant differences were observed in the mutation counts (**Supplementary Figure 3H**), no significant differences were found in the genomic alteration scores (**Supplementary Figure 3I**). Patients in the high-risk HRD score group showed higher mutation counts, indicating greater genomic instability.

### 3.7 Analysis of immune infiltration and immunomodulators based on HRD score

**Figure 6:**
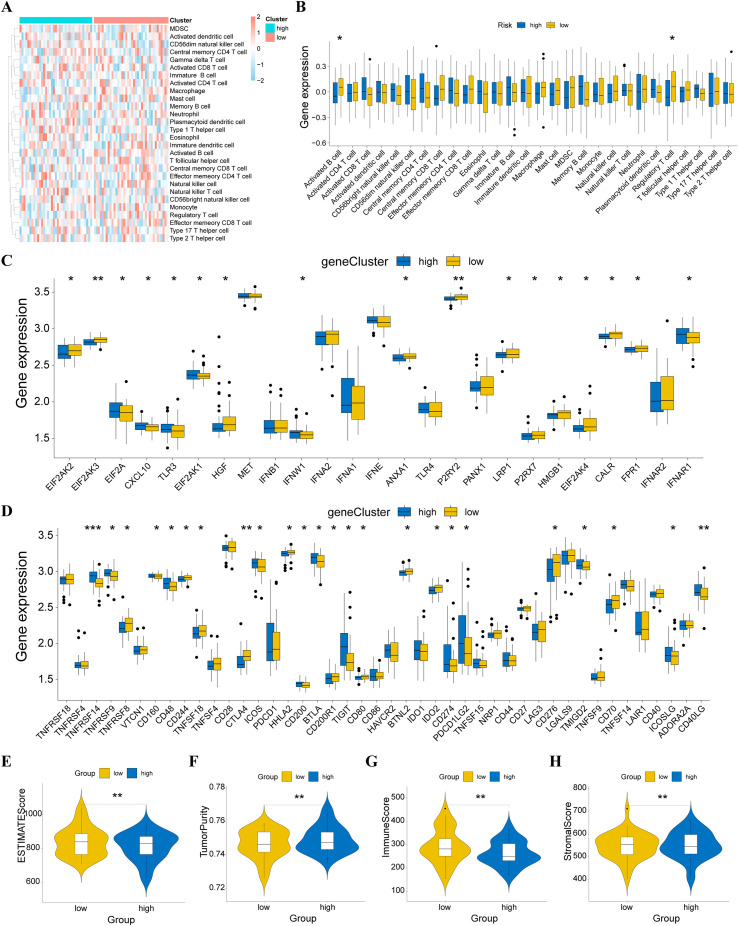
Characteristics of the immune microenvironment between high- and low-risk HRD groups. **(A)** Heat map of infiltration levels of 28 immune cells between HRD high and low-risk groups; **(B)** Box plot of differences in infiltration levels of 28 immune cells between HRD high and low-risk groups; **(C)** Box plot of differential ICD modulator expression between HRD high and low-risk groups; **(D)** Box plot of the differential ICP modulator expression between HRD high and low-risk groups; **(E)** Violin plot of ESTIMATE score differences; **(F)** Violin plot of tumor purity differences; **(G)** Violin plot of immune score differences; **(H)** Violin plot of stromal score differences. *p < 0.05, **p < 0.01, ****p < 0.001.

Immune cells are crucial in the development and progression of CHOL. We evaluated the distribution of 28 immune cell types using ssGSEA and compared their abundance to determine differences in immune cell infiltration between high- and low-risk HRD groups. Immune cell infiltration was more pronounced in the low-risk group (**Figures 6A, B**). Notably, active B cells and regulatory T cells, which are vital in tumor immunity, showed higher infiltration levels in the low-risk group.

We also evaluated the differential expression of ICP and ICD modulators between the two groups. Most ICD modulators were significantly up-regulated in the low-risk group (**Figure 6C**). Among ICP modulators, some genes remained significantly up-regulated in the low-risk group (**Figure 6D**). This suggests that low-risk patients may be more responsive to immunotherapy, while high-risk patients may develop immune-tolerant subtypes. The ESTIMATE analysis was performed to examine the relationship between tumor immune score, stromal score, and tumor purity in both groups. The ESTIMATE score was significantly higher in the low-risk group (**Figure 6E**), indicating a lower tumor purity (**Figure 6F**). Both the immune score (**Figure 6G**) and stromal score (**Figure 6H**) were also higher in the low-risk group, consistent with ssGSEA results. Overall, the low-risk HRD group exhibited higher immune infiltration, suggesting that these patients are more suitable for immunotherapy.

### 3.8 Immunotherapy analysis and identification of potential small molecule compounds

**Figure 7:**
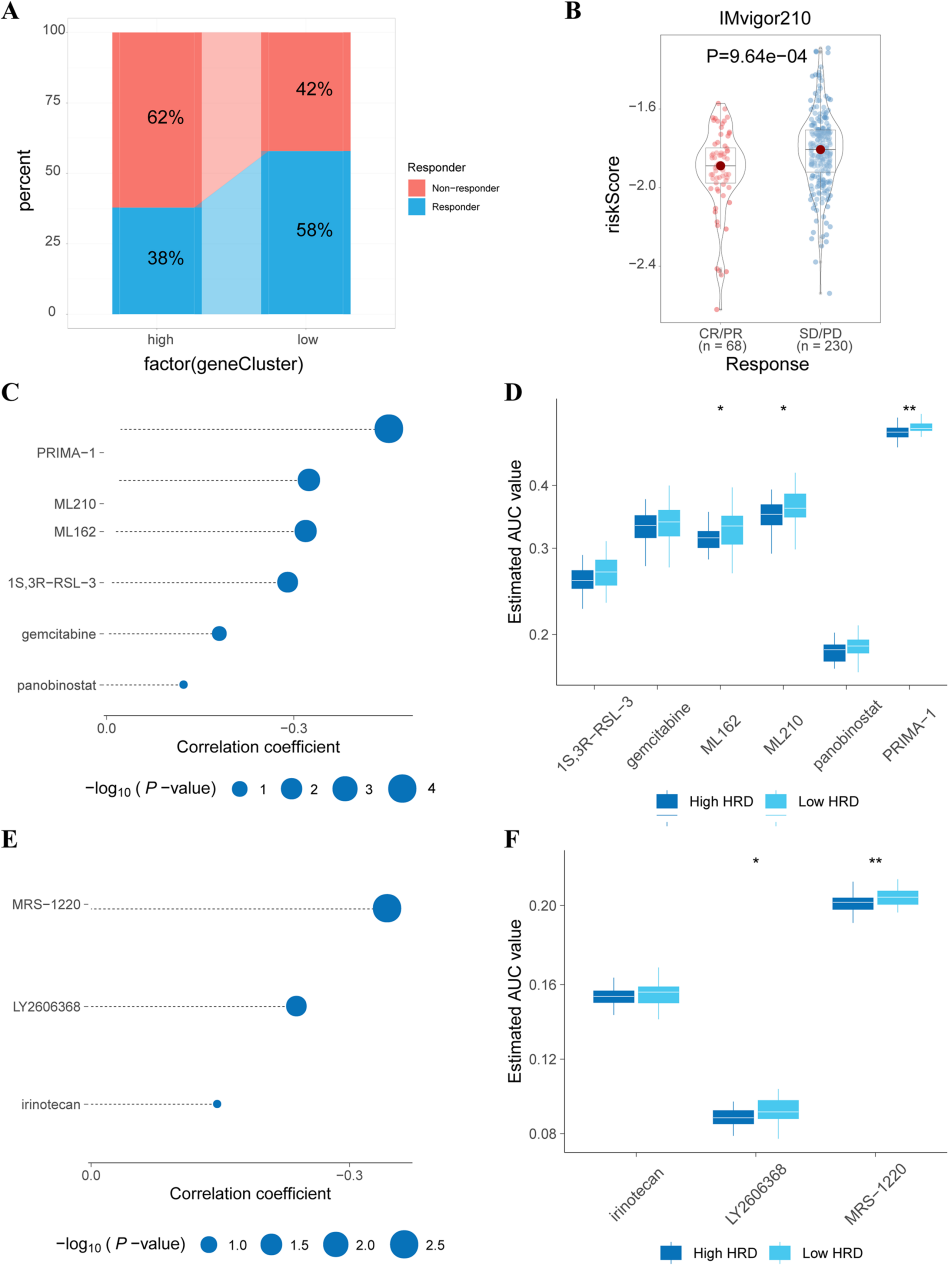
Immunotherapy and Drug-Sensitivity Analysis. **(A)** Bar graph of immune response differences between high and low-risk HRD groups predicted by TIDE; **(B)** Violin plot of efficacy differences based on the IMvigor210 bladder cancer immunotherapy cohort; **(C)** Lollipop plot of correlation coefficients and p-values between six small-molecule compounds and HRD scores based on CTRP drug-susceptibility data; **(D)** Box plot of estimated AUC values of six small-molecule compounds between high and low-risk groups based on CTRP drug susceptibility data; **(E)** Lollipop plot of correlation coefficients and p-values between three small molecule compounds and HRD scores derived from PRISM drug-susceptibility data; **(F)** Box plots of estimated AUC values of the three small-molecule compounds between high and low-risk groups based on PRISM drug susceptibility data. *p < 0.05, **p < 0.01.

To assess differences in immunotherapy responses between high- and low-risk HRD groups, we used the TIDE approach to predict immunotherapy responses, favoring the low-risk group (**Figure 7A**). To validate our results, we conducted differential analyses using the IMvigor210 bladder cancer immunotherapy dataset. The results indicated that the risk score was significantly lower in the CR/PR group, suggesting that low-risk patients are more likely to benefit from immunotherapy (**Figure 7B**).

To identify potential therapeutic drugs for CHOL patients, we used a comprehensive analysis combining CTRP and PRISM with Cmap. This analysis identified six CTRP-derived drugs (**Figure 7C**) and three PRISM-derived drugs (**Figure 7E**). The estimated AUC value of these drugs was negatively correlated with the HRD rating (**Figure 7D**) and was significantly lower in the high-risk groups (**Figure 7F**). Cross-referencing the CTRP and PRISM results, we identified two candidate compounds for the high-risk CHOL group: gemcitabine and panobinostat. The corresponding targets and mechanisms of action (MOA) are shown in **Supplementary Table 5**. The high sensitivity of CHOL patients to these compounds suggests their potential as therapeutic agents for the high-risk group.

### 3.9 Molecular docking

We performed molecular docking analysis using CB - Dock2 (https://cadd.labshare.cn/cb-dock2/php/index.php) to examine the interactions between potential drugs and receptor molecules. This analysis included target identification and visualization of the interactions (**Supplementary Figure 4**).

### 3.10 mRNA expression of differential genes in cancerous and adjacent tissues

In the prognostic model combining CoxBoost and LASSO machine-learning, ANXA2P1, BBOX1, KLHL33, MN1, OR51A4, and TRDN showed significant prognostic differences. We assessed the mRNA expression levels of these six genes in cancerous and adjacent tissues from six patients diagnosed with CHOL. RT-PCR results showed significantly higher mRNA expression of ANXA2P1, BBOX1, KLHL33, MN1, OR51A4, and TRDN in CHOL tissues compared to adjacent tissues (**Supplementary Figure 5**).

## 4 Discussion

CHOL is a highly aggressive malignancy originating from bile duct epithelial cells. It is classified into intrahepatic, perihilar, and distal types based on anatomical location (28). The incidence of CHOL has been increasing globally, accompanied by significant morbidity and mortality due to late diagnosis and limited treatment options (29). Surgical resection remains the primary curative treatment, but only a small percentage of patients are eligible for surgery at diagnosis (5). The high recurrence rate and resistance to conventional chemotherapy underscore the urgent need for novel therapeutic strategies and early diagnostic markers (6). The phenotypic heterogeneity of CHOL poses a significant challenge to its diagnosis and treatment. Recent studies have highlighted the potential of molecular profiling and genetic characterization to improve our understanding of CHOL pathogenesis and identify therapeutic targets (30). Identifying specific genetic mutations and alterations in signaling pathways has paved the way for targeted therapies and personalized medicine approaches (31). The development of predictive models based on HRD scores offers a promising avenue for enhancing CHOL treatment precision. HRD is associated with defects in DNA repair mechanisms, making tumors more susceptible to certain chemotherapeutic agents and PARP inhibitors (32). Our findings revealed a significant correlation between HRD scores and patient prognosis in CHOL. Specifically, patients with higher HRD scores exhibited poorer clinical outcomes, suggesting that HRD may serve as a prognostic indicator. Accurate assessment of HRD status can facilitate risk stratification and guide treatment decisions for CHOL patients. Furthermore, the HRD score holds promise as a valuable tool for predicting treatment responses and individualizing therapeutic strategies.

The detection of HRD scores could provide an optimal treatment plan for patients in the middle and late stages, and it has recently emerged as a topic of intensive research in tumor prevention and therapy. In CHOL, evaluating HRD scoring methods and developing prognostic models based on HRD have garnered significant interest. However, it is important to acknowledge that current HRD prognostic models have certain limitations in predicting tumor outcomes. Scheiter et al. identified alterations in HRR-related genes in a significant proportion of CHOL cell lines and clinical samples. The study employed the AmoyDx^®^HRD Focus Panel to determine genomic scar scores (GSS), which indicate HRD. Interestingly, although HRD-positive cell lines were identified, their sensitivity to the PARP inhibitor olaparib did not correlate well with HRD status, suggesting that additional factors may influence drug response in CHOL (33). This highlights the complexity of HRD as a biomarker and the need for further research to understand its role in therapeutic sensitivity. Identification of HRD-related genes is crucial for understanding the origins of HRD and for developing predictive biomarkers for CHOL. In this study, we employed a comprehensive approach involving 37 machine-learning algorithms to construct a multimachine learning model based on HRD feature genes. We identified the CoxBoost and LASSO algorithms as the optimal combination for creating a CoxBoost+ LASSO prognostic model. Using this model, we identified six genes (ANXA2P1, BBOX1, KLHL33, MN1, OR51A4, and TRDN) with significant differential expression.

BBOX1, as a carcinogenic long non-coding RNA, has been proven to possibly promote tumorigenesis and development through cell proliferation, migration and invasion, and its high expression is negatively correlated with prognosis (34). The MN1 gene is located in the region of 22q12 and plays an important role in posterior brain development and skull morphology. Study showed ABM-associated fusion proteins (MN1-BEND2 and MN1-CXXC5) induce overlapping transcriptional responses which leads to oncogenic transformation (35). Ou et al. found that ANXA2P1 is highly expressed in hepatocellular carcinoma and is associated with disease progression and poor prognosis. Knockdown of the gene can inhibit the growth, migration and invasion of HCC cells and reverse the EMT phenotype (36). There are relatively few reports on the KLHL33, OR51A4 and TRDN genes in tumor-related research. In 2018, a GWAS based on the Japanese population found that KLHL33 was a genetic susceptibility locus for Takayasu arteritis (TAK). TAK is an autoimmune disease involved in immune regulation between cells via signal pathway such as mTOR/JAK/STAT (37). Gilberto et al. found OR51A4 is hypomethylated in prostate cancer (38). Abnormalities in DNA methylation can affect cell function and promote the occurrence of cancer by inhibiting or activating genes. The proteins encoded by TRDN are involved in the excitation-contraction coupling of skeletal muscle. The mutation of this gene is associated with arrhythmia syndrome and sudden cardiac death. Studies have shown that when cholangiocarcinoma causes obstructive jaundice, inflammation and toxins will enter the heart through the systemic circulation, destroying myocardial cells, thereby inhibiting myocardial contractility and affecting left ventricular ejection function.

To validate these findings, we performed RT-PCR analysis on both tumor and adjacent non-tumor tissues and confirmed that the expression of these seven genes was significantly higher in tumor tissues. Survival analysis revealed significant prognostic differences associated with these genes. In addition, the HRD risk-scoring model demonstrated excellent predictive performance and emerged as an independent prognostic factor for patients with CHOL. Compared to other prognostic models, the HRD risk-scoring model exhibited higher stability. Our findings indicate that the CHOL-specific HRD risk scoring model is applicable not only to patients with CHOL alone but also to other tumor types such as osteosarcoma and hepatocellular carcinoma. By analyzing tumor data from these other cancer types, we found that our HRD risk-scoring model was similarly effective. In conclusion, our study addresses the limitations of current HRD scoring methods and prognostic models for CHOL. By constructing a multimachine learning model based on HRD feature genes and establishing a CoxBoost+ LASSO prognostic model, we identified significant genes associated with prognosis. The HRD risk-scoring model demonstrated robust predictive performance and stability, making it an effective and independent prognostic factor for patients with CHOL. Furthermore, our study highlights the broad applicability of the HRD risk-scoring model to other tumor types, underscoring its potential as a valuable biomarker in cancer research.

Genetic variations, including SNPs and CNVs, play crucial roles in the development and progression of cancers, including CHOL. Investigating these variations provides important insights into the underlying mechanisms of CHOL (39). HRD has shown promise in predicting the level of genetic alterations in CHOL. Furthermore, HRD has proven valuable for identifying gene mutations associated with drug sensitivity or resistance in CHOL (40). By analyzing the CHOL gene data, we can identify genetic alterations that may be relevant to drug responses. Our HRD risk score model revealed high mutation rates in specific genes, providing valuable information for personalized treatment strategies. This information can help identify potential therapeutic targets and aid in developing precision medicine approaches for CHOL.

In addition to genetic variations, HRD is associated with changes in signaling pathways in CHOL. The Mitogen-Activated Protein Kinase (MAPK) signaling pathway, Phosphatidylinositol 3-Kinase-AKT-mTOR (PI3K-AKT-mTOR) signaling pathway, Wnt signaling pathway, and Transforming Growth Factor-beta (TGF-β) signaling pathway plays important regulatory roles in the growth, proliferation, and differentiation of CHOL cells (33, 41–43). Our findings indicate that the high-score group in the HRD-related model had a higher percentage of gene mutations than the low-risk HRD group. Additionally, we identified the enrichment of mutation-prone genes in the RTK-RAS signaling pathway in CHOL patients, a critical cellular signaling pathway involved in cell growth, differentiation, survival, and proliferation. These findings provide valuable insights into the functional implications of HRD in CHOL and further support the critical role of HRD in the dysregulation of signaling pathways.

Cancer immunotherapy, represented by ICIs, has revolutionized the treatment of solid tumors. Previous studies have shown that ICPs and ICD modulators play important roles in the regulation of host antitumor immunity. Large cohort studies and randomized controlled trials have demonstrated that patients with dMMR/MSI-H cancers exhibit increased responses to immunotherapy and delayed disease progression (44). However, not all patients with dMMR cancers respond to immunotherapy due to variability in test assay sensitivity, and because dMMR/MSI-H are relatively rare (45). Moreover, effective biomarkers for predicting the efficacy of immunotherapy, aside from PD-1, PD-L1, MSI, and TMB, are limited. These ICI-associated biomarkers often fail to predict ICI treatment responses reliably. Interestingly, HRD can serve as a complementary biomarker to enhance diagnostic rates and improve the accuracy of predicting immunotherapy sensitivity, thereby reducing false negatives. Our study found that in the low-risk HRD group of CHOL patients, there was higher infiltration of B cells and regulatory T cells, along with upregulated expression of ICPs and ICD modulators. Immune infiltration is a critical step in tumor immunity and the regulation of host antitumor activity, making it a key factor in immunotherapy response. Our analysis indicates that the HRD score can evaluate immunotherapy response and predict the effectiveness of potential therapeutic drugs for CHOL.

We assessed the distribution of 28 immune cell types using ssGSEA and compared immune cell infiltration between high- and low-risk HRD groups. The low-risk HRD group showed significantly higher immune cell infiltration, ESTIMATE scores, and tumor purity. Using the TIDE method, we predicted immunotherapy responses between subgroups, finding significantly lower risk scores in the CR/PR groups. This suggests that genes associated with low HRD scores are more susceptible to immunotherapy. Additionally, we used CTRP and PRISM data to identify potentially sensitive drugs for the high-risk HRD group, with results indicating that gemcitabine is a promising therapeutic agent against CHOL. These findings underscore the complexity of the immune microenvironment in CHOL and highlight the potential of immunotherapeutic strategies targeting specific immune cell populations and pathways. By understanding the immune landscape and its impact on tumor progression and patient outcomes, more effective and personalized treatments for CHOL can be developed.

Although this study has achieved some promising findings, there are still several limitations. Firstly, this study is highly dependent on bioinformatics and computational analysis, without combining wet experimental verification. This may provide more support for the further verification of the results and the in-depth exploration of the mechanism. Secondly, the sample size, especially that of certain subgroups, is relatively small, which limits the universality of the research results. Thirdly, the lack of clinical validation means that the established predictive models and potential therapeutic targets have not yet been tested in clinical Settings. Furthermore, this study adopted the classic HRD scoring method (the unweighted sum of LOH, TAI and LST), while other methods such as GSS, HRDetect or assessment methods based on mutation characteristics may have different predictive capabilities. The comparative performance of these HRD assessment methods in CHOL has not been deeply explored yet. Furthermore, multiple datasets from different sources were used in the study, which may introduce batch effects and thereby affect the robustness of the results. Future related studies should optimize the HRD assessment strategy for CHOL patients by incorporating larger-scale, clinically validated cohorts, integrating computational analysis and experimental verification, and systematically comparing different HRD assessment methods, so as to make up for the deficiencies of this study.

## 5 Conclusion

In conclusion, we successfully developed a predictive model for CHOL based on HRD scores. Our study highlighted the prognostic value of six candidate genes, which might be powerful biomarkers for recurrence in CHOL survival, and suggested practical applications in prognostic predictions CHOL. The HRD score proved to be a robust predictor of prognosis, outperforming other reported biomarkers in multiple validation cohorts. Immunotherapy would benefit the low-risk HRD score patients, gemcitabine and panobinostat may be suitable for high-risk patients. This study also demonstrates the potential of HRD scores to predict outcomes in various cancers, including hepatocellular carcinoma, ovarian cancer, and osteosarcoma. Furthermore, we identified key signaling pathways and immune infiltration patterns associated with different HRD score groups, providing insights into underlying mechanisms. Potential therapeutic compounds were identified and validated using molecular docking, paving the way for future research and clinical applications aimed at improving personalized treatment strategies for patients with CHOL.

## Data Availability

The datasets presented in this study can be found in online repositories. The names of the repository/repositories and accession number(s) can be found in the article/**Supplementary Material**.
