# Supplementary material for: Identification of prognostic markers related to homologous recombination deficiency in cholangiocarcinoma using CoxBoost and LASSO machine learning techniques
Source: Front Immunol. 2026 Jan 21;17:1615657. doi: 10.3389/fimmu.2026.1615657 (PMC12867883; doi:10.3389/fimmu.2026.1615657)
Supplement: Supplementary file 1 [file Table1.docx]

Supplementary Material

# Supplementary Figures


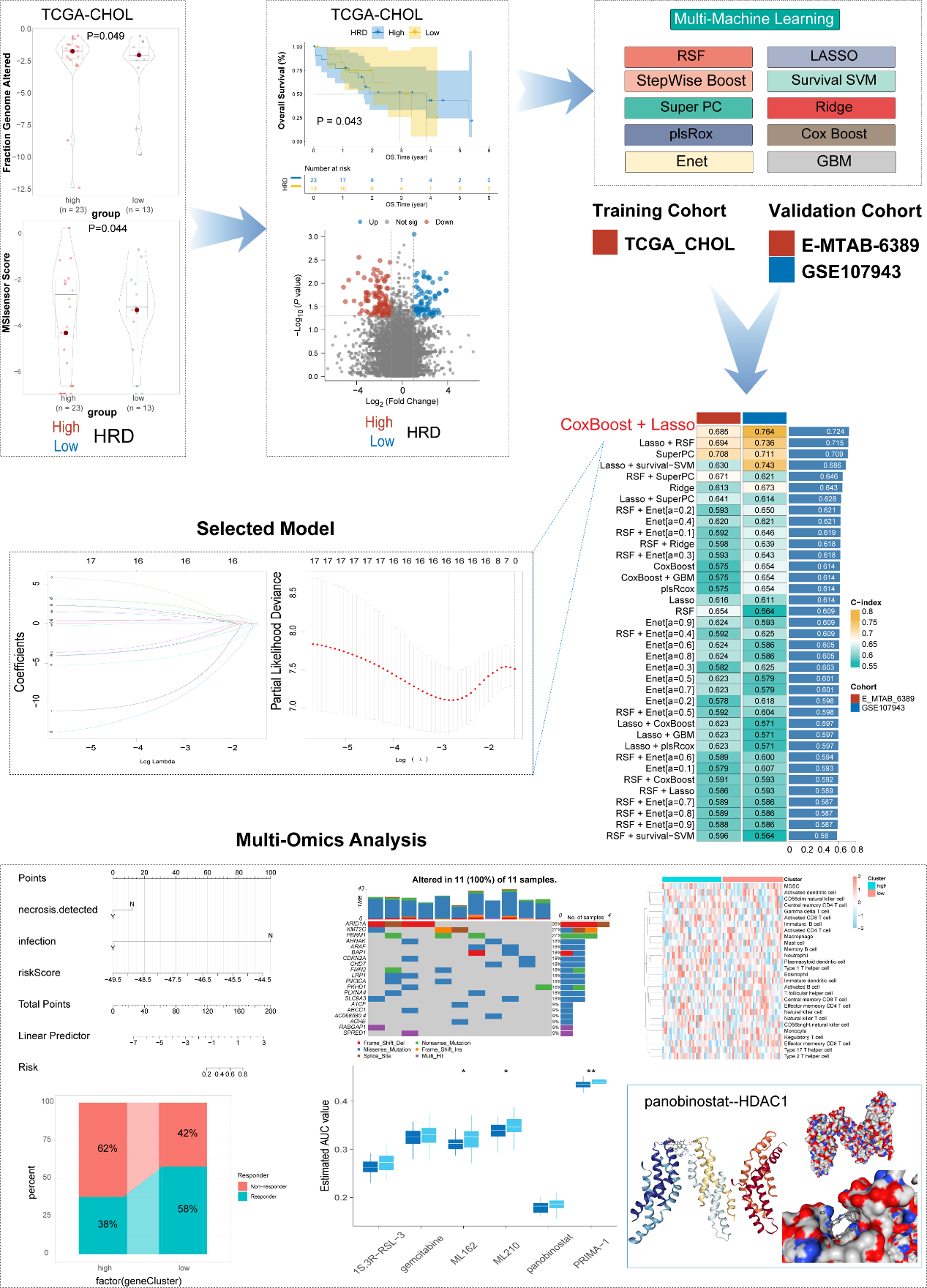


**Supplementary Figure 1**. The overall flow of this study. The process begins with obtaining and analyzing sample differences from the TCGA-CHOL dataset, followed by model selection and validation using various machine learning methods, ultimately employing multi-omics analysis to uncover potential biological mechanisms and therapeutic targets.


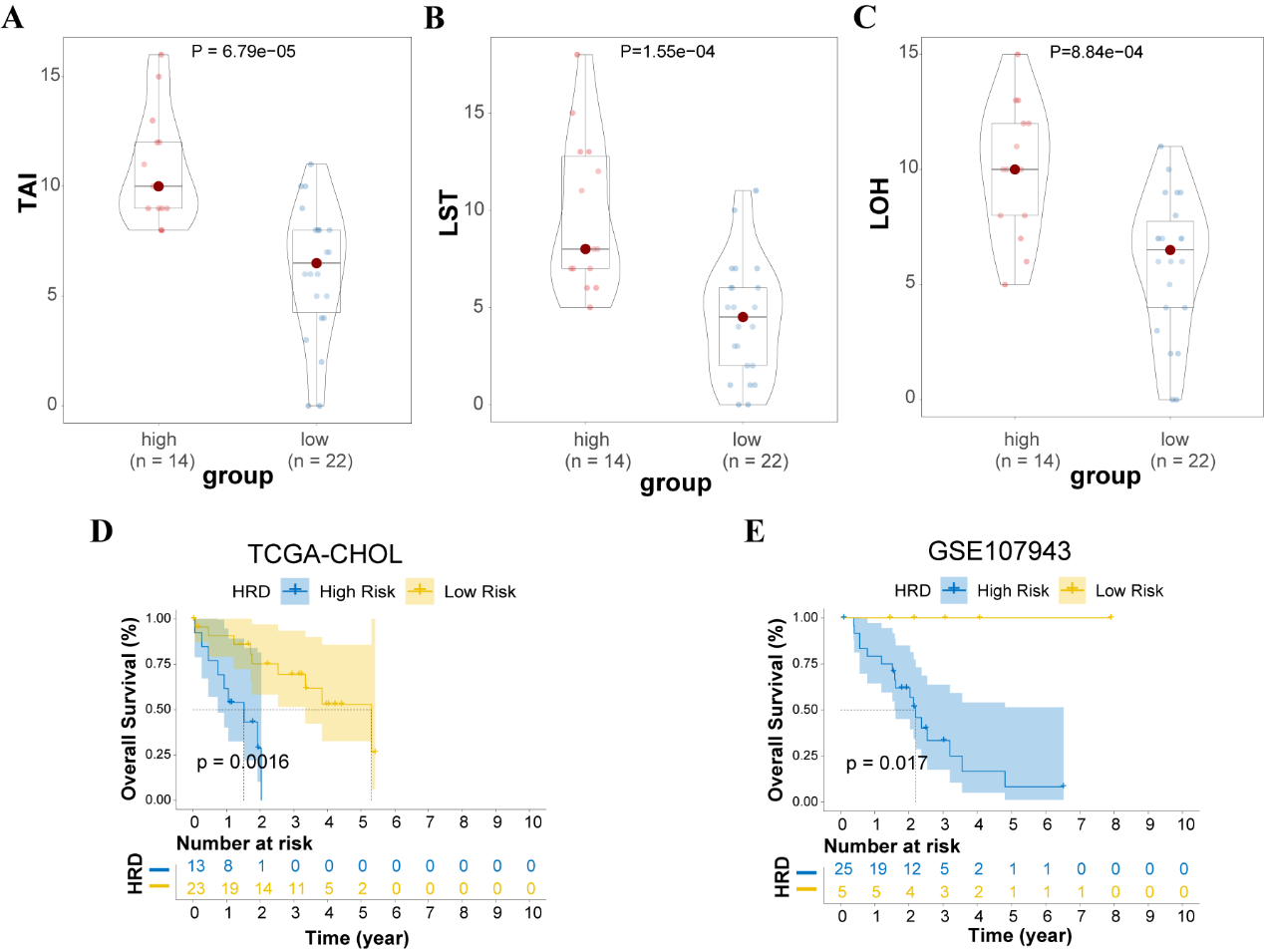


**Supplementary Figure 2**. Indicators of HRD and survival differences between high and low risk groups of HRD. A. Violin plot of Telomere Allelic Imbalance score differences between high and low HRD score groups in TCGA-CHOL patients; B. Violin plot of differences in Large Scale Transition Score between high and low HRD score groups in TCGA-CHOL patients; C. Violin plot of Loss of Heterozygosity Score between high and low HRD score groups in TCGA-CHOL patients; D. KM curves of patients in high and low risk groups in TCGA-CHOL dataset; E. KM curves of patients in the high and low risk groups of GSE107943.


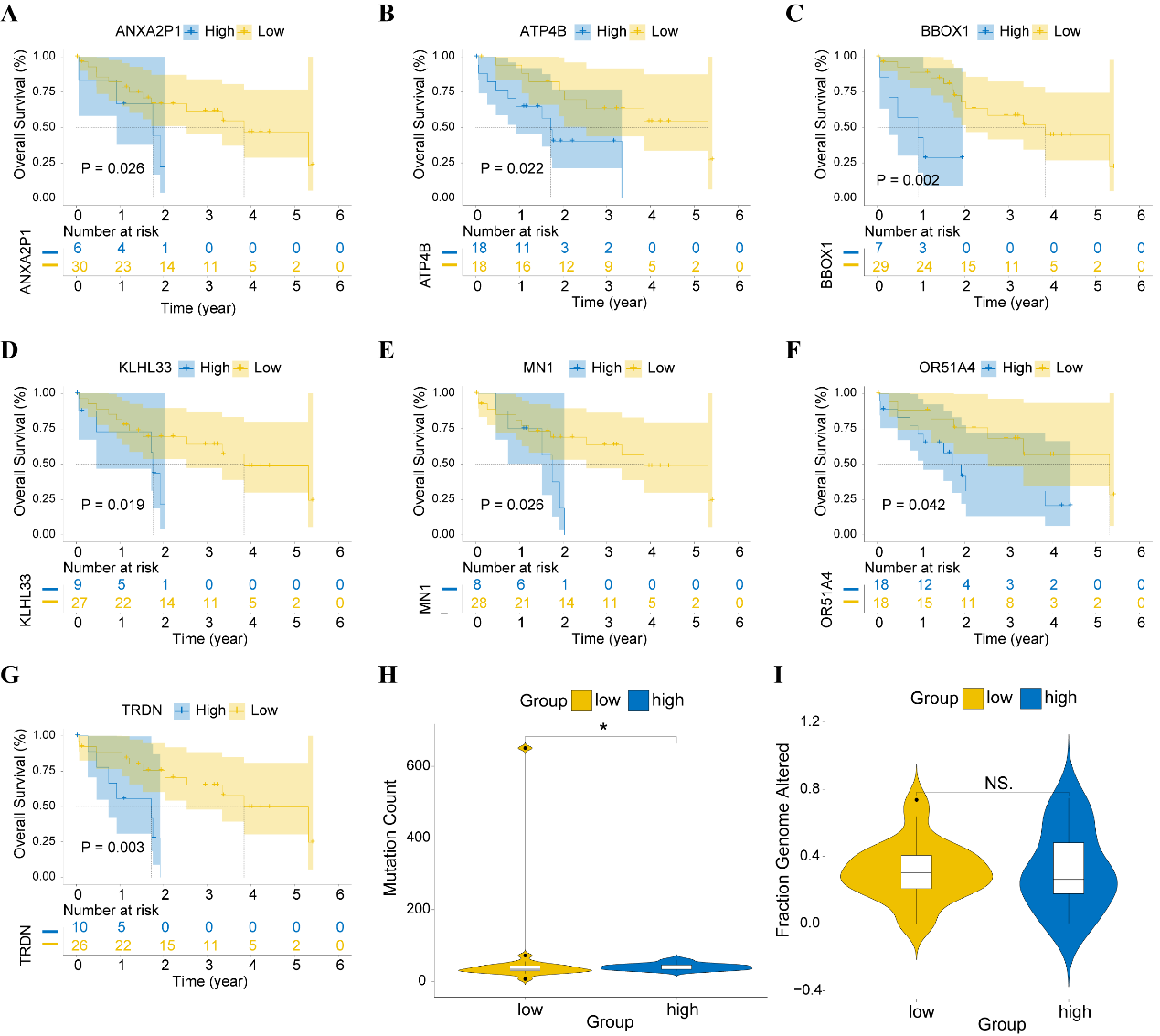


**Supplementary Figure 3**. Survival curves related to the expression of seven key genes and genomic differences between high and low HRD expression groups. A. Survival curve related to gene expression; B. ATP4B gene expression related survival curve; C. BBOX1 gene expression related survival curve; D. Survival curve related to KL33 gene expression; E. Survival curves related to MN1 gene expression; F. OR51A4 gene expression related survival curve; G. TRDN gene expression related survival curve; H. Violin plot of mutation count difference between high and low HRD expression groups; I. Violin plot of difference in genomic alteration scores between HRD high and low expression groups. * p < 0.05.


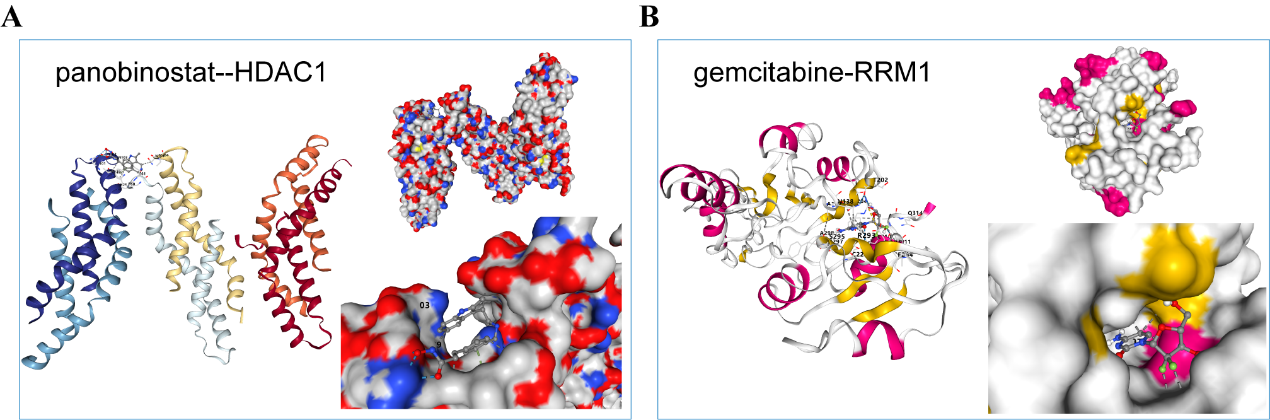


**Supplementary Figure 4**. Schematic diagram of molecular docking for the two candidate drugs and their receptor interaction pairs. A. Panobinostat interacting with the HDAC1 ligand-receptor; B. Gemcitabine interacting with the RRM1 ligand-receptor. The left side depicts the position of the compound and the receptor protein skeleton, while the right side shows detailed molecular docking of the ligand-receptor interaction pair.


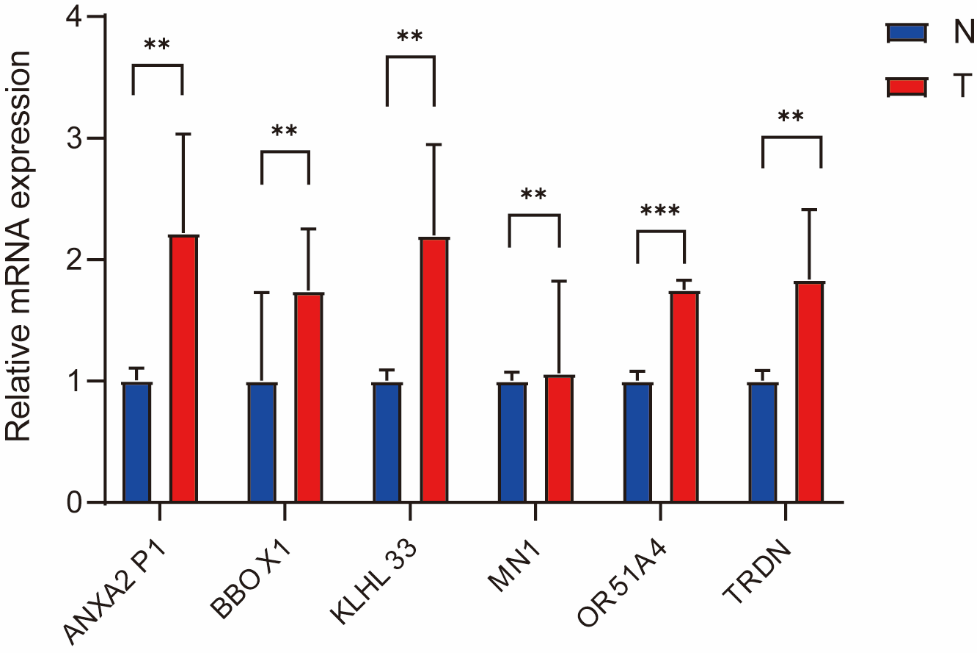


**Supplementary Figure 5**. mRNA expression of ANXA2P1, BBOX1, KLHL33, MN1, OR51A4, and TRDN in cholangiocarcinoma tissues and adjacent tissues. ** p < 0.01, **** p < 0.001.

# Supplementary Tables

**Supplementary Table 1**. Details of datasets used.

| Source | Samples | Tumor Type | Data type |
| --- | --- | --- | --- |
| E-MTAB-6389 | 75 | Cholangiocarcinoma | Expression profiling by array |
| GSE107943 | 30 | Cholangiocarcinoma | Expression profiling by array |
| IMvigor210 | 41 | Bladder Cancer | Expression profiling by array |
| TCGA-CHOL | 36 | Cholangiocarcinoma | RNA-seq |
| TCGA-LIHC | 363 | Liver Cancer | RNA-seq |
| TCGA-OS | 85 | Osteosarcoma | RNA-seq |

**Supplementary Table 2**. HRD score in TCGA-CHOL patients.

| Id | AI1 | LST1 | LOH | HRD | Cancer type |
| --- | --- | --- | --- | --- | --- |
| TCGA-W5-AA31-01A | 8 | 5 | 6 | 19 | Cholangiocarcinoma |
| TCGA-WD-A7RX-01A | 9 | 7 | 11 | 27 | Cholangiocarcinoma |
| TCGA-YR-A95A-01A | 7 | 7 | 9 | 23 | Cholangiocarcinoma |
| TCGA-W5-AA2Q-01A | 10 | 6 | 2 | 18 | Cholangiocarcinoma |
| TCGA-ZU-A8S4-01A | 8 | 8 | 8 | 24 | Cholangiocarcinoma |
| TCGA-ZD-A8I3-01A | 2 | 1 | 2 | 5 | Cholangiocarcinoma |
| TCGA-W5-AA39-01A | 6 | 2 | 4 | 12 | Cholangiocarcinoma |
| TCGA-W5-AA2X-01A | 11 | 3 | 9 | 23 | Cholangiocarcinoma |
| TCGA-3X-AAV9-01A | 10 | 8 | 12 | 30 | Cholangiocarcinoma |
| TCGA-ZH-A8Y1-01A | 8 | 7 | 7 | 22 | Cholangiocarcinoma |
| TCGA-3X-AAVB-01A | 8 | 5 | 10 | 23 | Cholangiocarcinoma |
| TCGA-4G-AAZT-01A | 5 | 1 | 5 | 11 | Cholangiocarcinoma |
| TCGA-3X-AAVA-01A | 3 | 1 | 4 | 8 | Cholangiocarcinoma |
| TCGA-ZH-A8Y6-01A | 0 | 0 | 0 | 0 | Cholangiocarcinoma |
| TCGA-W5-AA34-01A | 9 | 7 | 10 | 26 | Cholangiocarcinoma |
| TCGA-ZH-A8Y8-01A | 7 | 6 | 7 | 20 | Cholangiocarcinoma |
| TCGA-W5-AA2U-01A | 12 | 13 | 6 | 31 | Cholangiocarcinoma |
| TCGA-W5-AA2O-01A | 9 | 5 | 10 | 24 | Cholangiocarcinoma |
| TCGA-3X-AAVE-01A | 5 | 2 | 7 | 14 | Cholangiocarcinoma |
| TCGA-ZH-A8Y2-01A | 8 | 3 | 11 | 22 | Cholangiocarcinoma |
| TCGA-3X-AAVC-01A | 16 | 6 | 13 | 35 | Cholangiocarcinoma |
| TCGA-ZH-A8Y4-01A | 10 | 7 | 6 | 23 | Cholangiocarcinoma |
| TCGA-W6-AA0S-01A | 13 | 6 | 12 | 31 | Cholangiocarcinoma |
| TCGA-W5-AA2W-01A | 9 | 6 | 7 | 22 | Cholangiocarcinoma |
| TCGA-W5-AA2H-01A | 0 | 0 | 0 | 0 | Cholangiocarcinoma |
| TCGA-W5-AA30-01A | 11 | 12 | 15 | 38 | Cholangiocarcinoma |
| TCGA-4G-AAZO-01A | 8 | 13 | 5 | 26 | Cholangiocarcinoma |
| TCGA-W5-AA2T-01A | 8 | 5 | 9 | 22 | Cholangiocarcinoma |
| TCGA-ZH-A8Y5-01A | 12 | 15 | 10 | 37 | Cholangiocarcinoma |
| TCGA-W5-AA36-01A | 4 | 10 | 3 | 17 | Cholangiocarcinoma |
| TCGA-W5-AA33-01A | 4 | 11 | 8 | 23 | Cholangiocarcinoma |
| TCGA-W5-AA38-01A | 6 | 4 | 7 | 17 | Cholangiocarcinoma |
| TCGA-W5-AA2R-01A | 10 | 11 | 7 | 28 | Cholangiocarcinoma |
| TCGA-W5-AA2Z-01A | 6 | 4 | 6 | 16 | Cholangiocarcinoma |
| TCGA-W5-AA2I-01A | 9 | 7 | 8 | 24 | Cholangiocarcinoma |
| TCGA-W5-AA2G-01A | 15 | 18 | 13 | 46 | Cholangiocarcinoma |

**Supplementary Table 3**. List of RT-qPCR primers.

| Gene | Polarity | Sequences | Size (bp) |
| --- | --- | --- | --- |
| ANXA2P1 | Forward | 5’-TATTTCGGACACATCTGGTGACTTC-3’ | 73 |
|  | Reverse | 5’-GCCATCCTCTGCTCTTCTACCC-3’ |  |
| BBOX1 | Forward | 5’-TTCAAAACTTGGGAAAAGGATGG-3’ | 147 |
|  | Reverse | 5’-GGATGATGGAGGGCTGGATAAT-3’ |  |
| KLHL33 | Forward | 5’-AAATGCCAGGGAAGATGTAAAGAAG-3’ | 176 |
|  | Reverse | 5’-CCCCAAAGAACTCACTGCCAC-3’ |  |
| MN1 | Forward | 5’-GAACCCCAACAGCAAAGAAGCC-3’ | 190 |
|  | Reverse | 5’-AGCCACGAATGTCCCAAATCTG-3’ |  |
| OR51A4 | Forward | 5’-ATGTTGGCTATGTCAGACTTGGG-3’ | 209 |
|  | Reverse | 5’-GAGGTGTATCTCAGAGGGTTGTGG-3’ |  |
| TRDN | Forward | 5’-TGCCATCGTTATGTTTGATTTAGTG-3’ | 207 |
|  | Reverse | 5’-TCTCCTTTATCAGTATCTTCGTCACC-3’ |  |
| GAPDH | Forward | 5’-GAAGGTGAAGGTCGGAGTC-3’ | 227 |
|  | Reverse | 5’-GAAGATGGTGATGGGATTTC-3’ |  |

**Supplementary Table 4**. Coeffiences of selected 16 genes for model construction.

| Gene | Coef |
| --- | --- |
| CD1D | -7.218264704 |
| KCNH1 | 0.673887128 |
| HTR3D | 2.872045352 |
| ANXA2P1 | 0.72528429 |
| TRDN | -1.454926304 |
| ZC3HAV1 | -2.027837572 |
| ENTPD2 | 0.978635433 |
| OR51A4 | 1.875151526 |
| BBOX1 | -8.272817436 |
| CCDC59 | 1.428966269 |
| ATP4B | 0.133739909 |
| KLHL33 | 1.03445102 |
| DHDH | 0.425239186 |
| HAO1 | 1.711818266 |
| IGLV4-3 | -2.176771896 |
| MN1 | -3.668535138 |

**Supplementary Table 5**. Selected Therapeutic Drugs, Targets, and Mechanisms of Action in the Cmap Database.

| Score | ID | Name | Description | Target | MOA |
| --- | --- | --- | --- | --- | --- |
| 44.54 | BRD-K15108141 | gemcitabine | Ribonucleotide reductase inhibitor | RRM1, CMPK1, RRM2, TYMS | Ribonucleotide reductase inhibitor |
| 31.51 | BRD-K02130563 | Panobinostat | HDAC inhibitor | HDAC1, HDAC2, HDAC3, HDAC4, HDAC6, HDAC7, HDAC8, HDAC9 | HDAC inhibitor |
